# Intrathecal chloroprocaine or hyperbaric prilocaine for ambulatory knee surgery? A prospective randomized study

**DOI:** 10.1186/s40634-021-00332-3

**Published:** 2021-02-24

**Authors:** E Guntz, C Vasseur, D Ifrim, A Louvard, J F Fils, Y Kapessidou

**Affiliations:** 1 Department of Anesthesiology, Hôpital Braine L’Alleud Waterloo, Université Libre de Bruxelles (ULB), 35 rue Wayez, 1420 Braine l’Alleud-Waterloo, Belgium; 2 Independant Biostatistician – Ars Statistica, Nivelles, Belgium; 3 Department of Anesthesiology, CHU St Pierre, ULB, 322 rue Haute, 1000 Bruxelles, Belgium

**Keywords:** Spinal anesthesia, Hyperbaric prilocaine, Chloroprocaine, Prospective study, Sensory block

## Abstract

**Purpose:**

The aim of this study was to compare intrathecal 1% chloroprocaine with 2% hyperbaric prilocaine in the setting of ambulatory knee arthroscopy. We hypothesized that complete resolution of the sensory block was faster with chloroprocaine.

**Methods:**

Eighty patients scheduled for knee arthroscopy were included in this prospective randomized double-blind study. Spinal anesthesia was performed with either chloroprocaine (50 mg) or hyperbaric prilocaine (50 mg). Characteristics of sensory and motor blocks and side effects were recorded.

**Results:**

Mean time to full sensory block recovery was shorter with chloroprocaine (169 (56.1) min *vs* 248 (59.4)). The characteristics of the sensory blocks were similar at the T12 dermatome level between the two groups. Differences appeared at T10: the percentage of patients with a sensory block was higher, onset quicker and duration longer with hyperbaric prilocaine. The number of patients with a sensory block at T4 dermatome level in both groups was minimal. Times to full motor recovery were identical in both groups (85 (70–99) *vs* 86 (76–111) min). Time to spontaneous voiding was shorter with chloroprocaine (203 (57.6) min *vs* 287.3 (47.2) min). Incidence of side effects was low in both groups.

**Conclusions:**

When considering the characteristics of the sensory block, the use of chloroprocaine may allow an earlier discharge of patients. Cephalic extension was to a higher dermatomal level and the sensory block at T10 level was of prolonged duration with hyperbaric prilocaine, suggesting that the choice between the two drugs should also be performed based on the level of the sensory block requested by the surgery.

This study is registered in the US National Clinical Trials Registry, registration number: NCT030389, the first of February 2017, Retrospectively registered.

## Introduction

Anesthesiologists have conducted several studies in order to adapt the characteristics of intrathecal local anesthetics to the length of surgeries. Hyperbaric lidocaine and mepivacaine have been used but due to the high risk of transient neurological symptoms (TNS), spinal administration of these local anesthetics has been abandoned [1, 2]. Articaine has also been promoted but it seems that its neurological safety should be established before further promotion in routine practice [3]. During the last decade, new formulations of plain chloroprocaine and hyperbaric prilocaine have gained interests as short and intermediate-acting spinal anesthetics respectively. In order to allow clinicians to choose between these two local anesthetics and fine-tune their spinal anesthesia according to the duration and the level of the sensory block requested by the surgery, we conducted a prospective double-blind randomized study comparing these 2 local anesthetics for patients undergoing ambulatory knee arthroscopy. We hypothesized that the complete resolution of the sensory block would be different after injection of chloroprocaine compared to hyperbaric prilocaine. Secondly, we compared the onset, the level of the sensory block and the duration of the motor block of the two drugs.

## Materials and methods

This study was approved by the local Medical Ethics Committee (Centre Hospitalier Universitaire Saint Pierre, Université Libre de Bruxelles (ULB) Bruxelles, Chairperson Dr. E. Stevens. Research Ethics Board number: code EC 332, OM 157; date of protocol approval: 14 of April 2016; protocol number: B076201627870) and registered in the US National Clinical Trials Registry (registration number: NCT030389). After written informed consent, patients meeting the following criteria were enrolled in this study: American Society of Anesthesiologists (ASA) physical status I-III, aged 18–80 years, body mass index (BMI) 20–30 kg/m^2^, height 155–190 cm, and scheduled for day-case knee arthroscopy under spinal anesthesia. Exclusion criteria were standard contraindications to neuraxial block and patient refusal. Patients were randomized according to a computer-generated allocation sequence in 2 groups: the chloroprocaine group and the hyperbaric prilocaine group. The study was double blinded.

Midazolam 1 mg *iv* was administered as a premedication and patients received Ringer’s lactate solution 500 mL *iv* throughout the entire operation. Continuous electrocardiography and pulse oximetry (SpO2) were applied to each patient, noninvasive arterial blood pressure was measured every three minutes during the procedure.

Spinal anesthesia was performed in the sitting position under aseptic conditions using the midline approach at the L3-L4 interspace with a 25G Whitacre needle (Becton Dickinson, Madrid, Spain). Immediately after injection of 50 mg of 2% hyperbaric prilocaine or 50 mg of 1% chloroprocaine (Sintetica SA, 6850 Mendrisio, Switzerland) patients laid supine in the neutral position. Sensory and motor blockade were assessed 5, 10, 20, and 30 min after intrathecal injection. Pinprick (needle of a Dejerine reflex hammer, Neurologicals 5038) and cold tests were used to evaluate the level of sensory block. The Bromage scale was used to evaluate the motor block (0:no motor block; 1:hip blocked; 2:hip and knee blocked; 3:hip, knee, and ankle blocked). The tourniquet was inflated to a pressure of 340 mmHg for each patient when the sensory block was achieved at T12 dermatome level.

Pain was assessed using a 10-cm horizontal visual analogue scale (VAS). When inadequate analgesia occurred (VAS > 2 after inflation of the tourniquet or after incision) a continuous *iv* infusion of remifentanil was administered, general anesthesia with a laryngeal mask was provided if requested and spinal anesthesia was recorded as a failure.

Hypotension (defined as a 20% drop in systolic blood pressure) and bradycardia (variations of 20% below the baseline) were treated with ephedrine 5–10 mg or atropine 0.5 mg *iv* at the discretion of the attending anesthesiologist.

After surgery, patients’ follow-up continued in the postanesthesia care unit every ten minutes until complete recovery of motor block. Resolution of the sensory block was recorded when all the sensory tests were negative and patients declared regaining full sensation. At this time, the patient was considered eligible for home discharge. Overall, the blinded investigator recorded the following variables:

- 1. duration of the sensory block.
- 2. characteristics of the sensory blocks at dermatome levels T12, T10, T4: percentage of patients at this level, onset, duration.
- 3. onset, duration and level of motor block.
- 4. side effects including hypotension, bradycardia or urinary retention (incapacity to void after complete resolution of the blocks).
- 5. For the first 30 days after surgery, patients were asked to report any postoperative problems to the anesthesiologist involved in the study.

### Statistics

The chi-squared test was used to investigate differences between discrete variables. For continuous variables, the two assumptions of the *t*-test were checked (normality of the residuals and homogeneity of the variances, results not showed). If both assumptions were met a *t*-test was performed (means and standard deviations are reported). If at least one of the two assumptions was not met, a Wilcoxon signed rank test was performed (medians and inter-quartile ranges by group are reported). For longitudinal data, generalized estimating equations were used to model the relationship of discrete parameters through time [4]. In each model, we tested whether an increase in probability was observed, whether group differences were observed and whether an interaction effect between groups and times was observed. The R software (R Core Team, 2016, R 3.2.2. for Windows; R Foundation for Statistical Computing, Vienna, Austria: https://www.R-project.org/), was used to produce statistical results. A sample size calculation for the primary outcome was performed based on the time between the spinal anesthesia and the complete resolution of the sensory block. With the following parameters: an alpha equal to 0.05, a power of 90%, standard deviation equal to 60, a difference between the two groups of 50 with at least an observed difference of 10 and an equal group allocation was required. Based on these values, a sample size of 39 patients in each group was necessary in order to observe the retained difference between the two groups.

## Results

**Fig. 1:**
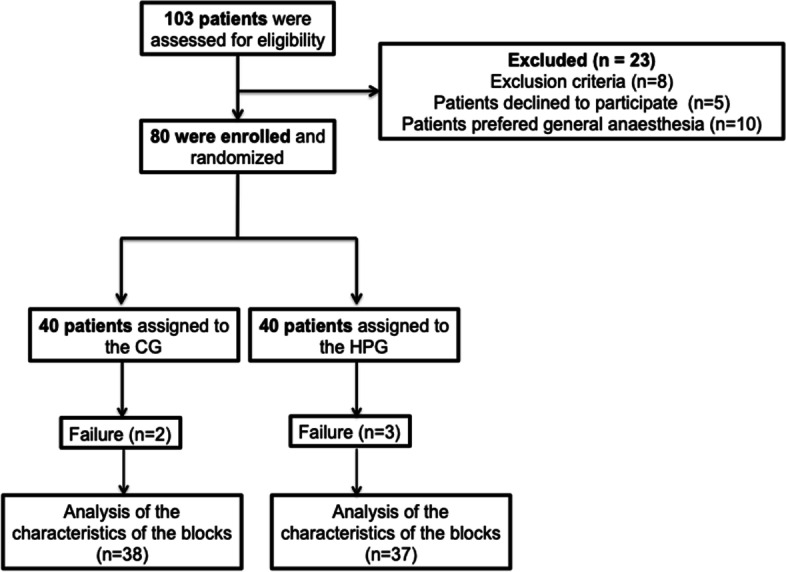
Flowchart patients scheduled for knee arthroscopy with Chloroprocaine (CG) or Hyperbaric prilocaine (HPG)

Patients were recruited between May 2016 and March 2017. 103 patients scheduled for knee arthroscopy were selected during the preanesthetic assessment, 23 declined to participate in the study. Two groups of 40 patients were recruited for the study (Fig. 1). The two groups were similar in terms of demographic data. 2 patients in the chloroprocaine group and 3 patients in the hyperbaric prilocaine group required analgesic supplementation.

Mean time to full sensory block recovery, the primary outcome, was 79 min shorter for the patients in the chloroprocaine group compared to those in the hyperbaric prilocaine group (Table 1).

**Table 1:** Time to full sensory block recovery, time to first spontaneous voiding and side effects. Mean (standard deviation) and absolute numbers

	Chloroprocaine	Prilocaine	*P*-value
Time to full sensory block recovery	169 (56.1)	248 (59.4)	< 0.001
Time to first spontaneous voiding	203 (57.6)	287.3 (47.2)	< 0.001
Urinary retention: catheterisation	0	0	/
Bradycardia	2	3	0,802
Hypotension	0	2	0,329

**Fig. 2:**
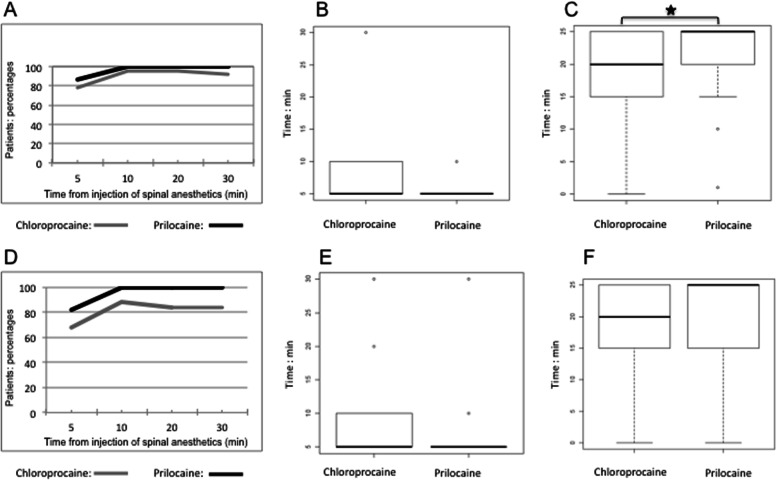
**a** Percentage of patients with a complete loss of cold sensation at the T12 dermatome during the first 30 min after local anesthetics injection. **b** Time to obtain a T12 dermatome level extension of sensory block evaluated with cold test. **c** Duration of sensory block at T12 dermatome level evaluated with cold test. **d** Percentage of patients with negative pin-prick test at T12 dermatome level during the first 30 min after local anesthetics injection. **e** Time to obtain a T12 dermatome level extension of sensory block evaluated with pin-prick test. **f** Duration of sensory block at T12 dermatome level evaluated with pin-prick test. Statistically significant differences are marked with *(*P* < 0.05)

The percentage of patients with a complete loss of cold sensation at the T12 dermatome level was similar in both groups: after 10 min a plateau was achieved, with 94.90% in the chloroprocaine group vs 100% in the hyperbaric prilocaine group (*p* = 0.49). Identical results were recorded after 20 and 30 min (Fig. 2a). The pin-prick test provided a similar profile (Fig. 2d).

Median times (min) to reach T12 dermatome level were similar in both groups (cold test: 5 (5–10) *vs* 5, (5–5); W = 832.5, *p*-value = 0.105. Pin-prick test: 5, (5–10) *vs* 5, (5–5) W = 811, *p*-value = 0.23) (Fig. 2b, e).

The pin-prick test did not reveal any difference in the duration (min) of sensory blocks at T12 dermatome level (20 (15–25) *vs* 25 min, (20–25), W = 566, *p*-value = 0.08). In contrast to this, the cold test showed a longer median duration of sensory block at T12 dermatome level with hyperbaric prilocaine compared to chloroprocaine (25 (20–25) *vs* 20 (15–25); W = 534.5, *p*-value = 0.03) (Fig. 2c).

**Fig. 3:**
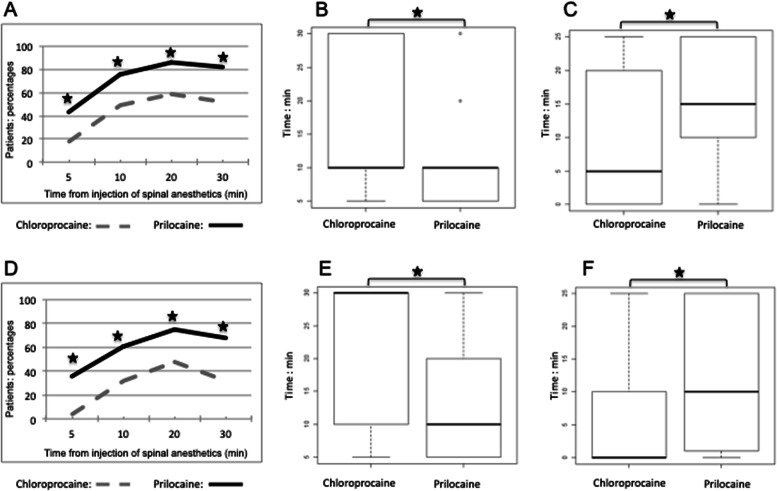
**a** Percentage of patients with a complete loss of cold sensation at T10 dermatome during the first 30 min after local anesthetics injection. **b** Time to obtain a T10 dermatome level extension of sensory block evaluated with cold test. **c** Duration of sensory block at T10 dermatome level evaluated with cold test. **d** Percentage of patients with negative pin-prick test at T10 dermatome during the first 30 min after local anesthetics injection. **e** Time to ontain a T10 dermatome level extension of sensory block evaluated with pin-prick test. **f** Duration of sensory block at T10 dermatome level evaluated with pin-prick test. Statistically significant differences are marked with *(*P* < 0.05)

During the first 30 min after local anesthetics injection, the percentage of patients with complete loss of cold sensation and a negative pin-prick test at the T10 dermatome level was higher with hyperbaric prilocaine. A peak was achieved after 20 min in both groups with the cold test (59% in the chloroprocaine group vs 86.10% in the hyperbaric prilocaine group, *p* = 0.18) and the pin-prick test (48% in the chloroprocaine group vs 75% in the hyperbaric prilocaine group, *p* = 0.11) (Fig. 3ad).

Median time (min) to reach this level based on the cold test and the pin-prick test was superior with chloroprocaine (cold test: 10 (10–30) *vs* 10 (5–10); *p*-value = 0.02. Pin-prick-test: 30 (10–30) *vs* 10 (5–20); *p*-value < 0.001) (Fig. 3b.e).

The median duration (min) of sensory block at T10 dermatome level evaluated with the cold test and the pin-prick test was longer with hyperbaric prilocaine (cold test: 15 (10–25) *vs* 5 (0–20); *p*-value = 0.002; pin-prick-test: 10 min, (1–25) *vs* 0 min, (0–10); *p*-value < 0.001) (Fig. 3c.f).

**Fig. 4:**
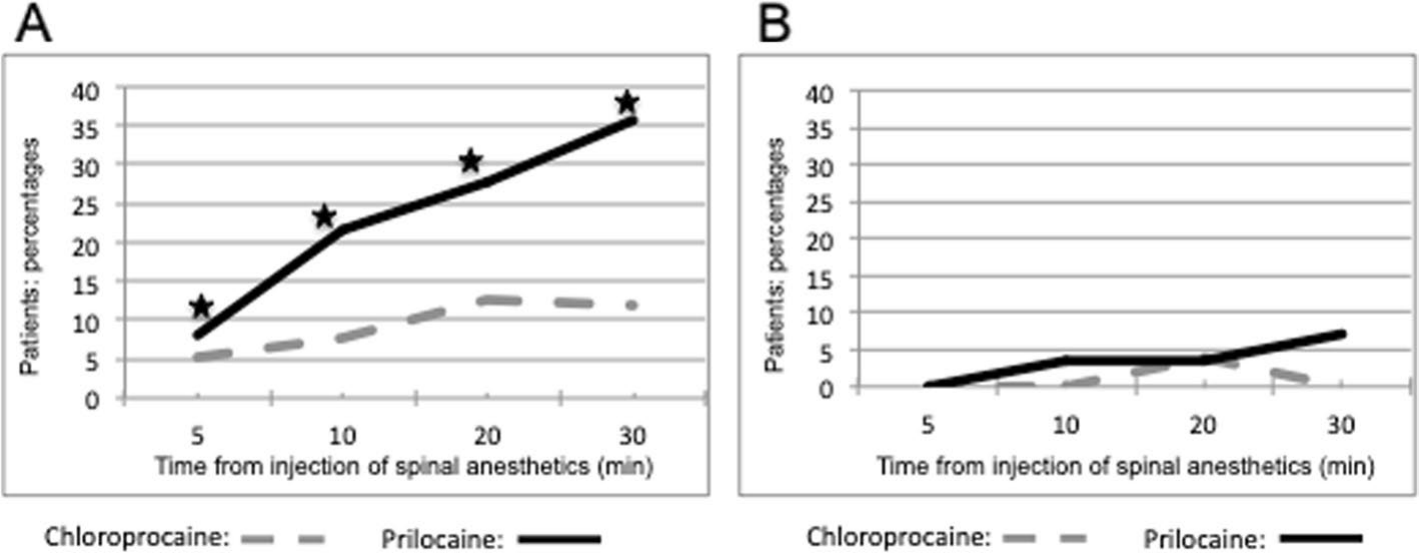
**a** Percentage of patients with a complete loss of cold sensation at T4 dermatome level during the first 30 min after local anesthetics injection. **b** Percentage of patients with negative pin-prick test at the T4 dermatome level during the first 30 min after local anesthetics injection. Statistically significant differences are marked with *(*P* < 0.05)

During the first 30 min after local anesthetic injection, the percentage of patients with a complete loss of cold sensation at the T4 dermatome level was greater in the hyperbaric prilocaine group compared to the chloroprocaine group. After 30 min cold sensation was absent for 35.71% in the patients of the hyperbaric prilocaine group compared to 12% of the patients of the chloroprocaine group (*p* < 0.001) (Fig. 4a). Only 4% of the patients in the chloroprocaine group exhibited a negative pin-prick test after 20 min, 0% after 30 min. Similarly, in the hyperbaric prilocaine group, a negative pin-prick test was recorded for 3.57% of the patients after 20 min and 7.14% after 30 min (*p* > 0.05) (Fig. 4b).

**Fig. 5:**
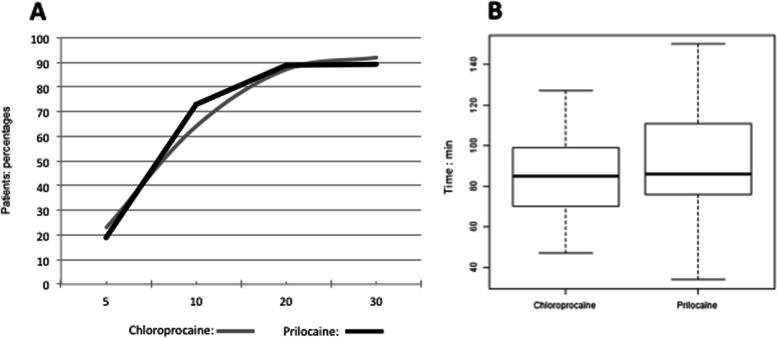
**a** Percentage of patients with a complete motor block (Bromage 3) during the first 30 min after local anesthetics injection. **b** Time to obtain complete recovery of motor block (Bromage 0). Statistically significant differences are marked with *(*P* < 0.05)

The percentage of patients with a complete motor block was comparable between groups during the first 30 min. Bromage 3 was recorded for 92% of the patient with chloroprocaine compared to 89.30% of the patient with hyperbaric prilocaine after 30 min (*p* = 1.00) (Fig. 5a). No difference in median duration of motor block was recorded between groups: 85 (70–99) min with chloroprocaine, 86 (76–111) min with hyperbaric prilocaine, *p* = 0.24 (Fig. 5b).

Time to first spontaneous voiding was shorter with chloroprocaine. Urinary retention and TNS were absent in both groups, hypotension was absent in the chloroprocaine group. Failures and bradycardia were rare (Table 1). No complications were recorded during the first 30 postoperative days.

## Discussion

Our results showed that time to full recovery of the sensory block was shorter with chloroprocaine compared with hyperbaric prilocaine whilst duration of motor blocks was similar*.*

Both local anesthetics provided a stable T12 sensory block in a high percentage of patients. At T10 dermatome level this percentage was lower in both groups but greater in the hyperbaric prilocaine group when compared with the chloroprocaine group. Neither chloroprocaine nor hyperbaric prilocaine were not able to produce a surgical block at the T4 dermatome level. Onset and duration of the sensory block were similar at T12 dermatome level, except when the duration was assessed with the cold test. Differences appeared with both sensory tests at T10 dermatome level: the time of onset of the sensory block was shorter while the duration of the block was longer with intrathecal hyperbaric prilocaine. Time to first spontaneous voiding was longer in the hyperbaric prilocaine group.

The mean time to obtain complete resolution of the sensory block was one and a half hours shorter with chloroprocaine when compared with hyperbaric prilocaine.

When compared to articaine, the complete resolution of the sensory block was obtained 30 min earlier with chloroprocaine [5]. Moreover, articaine, compared to hyperbaric prilocaine may result in a faster discharge [6]. In this context, our results bring additional supports and clarifications to the pre-existing classifications of local anesthetics for spinal anesthesia [7, 8] Interestingly duration of motor block was similar in both groups. Therefore, chloroprocaine is able to promote a shorter sensory block compared to hyperbaric prilocaine with a similar motor block which can be helpful for the performance of ambulatory surgery.

A high percentage of patients exhibit a sensory block at T12 dermatome level in the 2 groups. This percentage decreases at T10 dermatome level: at this level the percentage of patients exhibiting a sensory block is greater with hyperbaric prilocaine. Förster et al*.* recorded 95% of sensory block at L1 dermatome level after intrathecal injection of 40 mg of chloroprocaine, whilst 80% of patients reached the T10 dermatome level. Maximal extension of the sensory block ranged between T10 and T7 dermatome level. In a separate study, they recorded 92% of sensory block at L1 dermatome level with 40 mg of chloroprocaine and a sensory block at T10 dermatome level for 64% of the patients; maximal extension of the sensory block ranged between T6 and T12 dermatome level [5, 9] Casati et al*.* described the maximal extension of the sensory block ranging between T12 and T7 with 50 mg of chloroprocaine [10]. In a previous study we recorded the maximal extension of the sensory block with hyperbaric prilocaine between T12 and T4 dermatome level [11]. Manassero et al*.* recorded this peak between T11 and T4 with 50 mg of hyperbaric prilocaine [12]. 60 mg of hyperbaric prilocaine was described as able to provide a maximal extension of the sensory block to the T6 dermatome level [13]. These results and our data highlight that intrathecal injection of 50 mg of chloroprocaine or hyperbaric prilocaine is able to provide a sensory block for more than 90% of the patients at the T12 dermatome level. The situation is different at the T10 dermatome level. At this level the percentage of patients experiencing a sensory block (less than 80% with hyperbaric prilocaine, 60% with chloroprocaine) started to decrease after 20 min. The maximal extension of the sensory block rarely reaches T4 dermatome level: in our study, sensory blocks recorded at this level were minimal.

Altogether our results suggest that the choice of local anesthetics should be based on both the duration and the required dermatomal level of the surgical procedure. Indeed, as mentioned above, local anesthetics employed for spinal anesthesia have been defined as short, intermediate and long acting [7, 14]. This classification is very useful to choose between local anesthetics in regard to the duration of the procedure [15]. Based on our results we propose to add the dermatomal level of the surgery as a criterion determining the choice of the drug. Short surgical procedures performed under T12 dermatome level should be performed with chloroprocaine. Intermediate procedures performed under T12 should be performed with hyperbaric prilocaine. Short and intermediate procedures between T12 and T10 should be performed with hyperbaric prilocaine. Above T12, hyperbaric prilocaine is certainly the choice for short procedures.

The choice of the local anesthetic can be judicious, yet discharge of the patient can be impaired by side effects like urinary retention or delayed micturition. In the present study we did not record any urinary complication with either local anesthetic. Kreutzinger et al*.* described 23% of bladder catheterization after injection of 60 mg of intrathecal hyperbaric prilocaine, this percentage decreases to 8.3% with 50 mg [6, 16]. Other studies did not describe any urinary retention after intrathecal injection of 60 mg hyperbaric prilocaine [13, 17, 18].^.^ In Kreutzinger’s study, oral intake was allowed up until 2 h prior to surgery and the mean fluid administration during the procedure was 1291 ml [16]. In all the other cited studies and the present work, the mean fluid administration was inferior. The incidence of urinary retention with chloroprocaine is very low: Hejtmanek et al*.* reported 19 urinary retentions with 503 patients after intrathecal injection of chloroprocaine. Interestingly two third of these patients had procedures that increase the risk of urinary retention; this was not the case in our study [19]. Moreover, a 500 ml pre-load seems safe for chloroprocaine in regard to bladder filling [20].

Mean time to first spontaneous voiding is less than the mean time to obtain complete resolution of the sensory block in both groups and 80 min shorter with chloroprocaine. Nevertheless, spontaneous micturition as a criterion for discharge after short and intermediate spinal anesthesia is still debated and recommended for selected patients only [21].

Interestingly, the cold test was positive in the hyperbaric prilocaine group for more than a third of the patients at T4 dermatome level during the first 30 min. Therefore, the occurrence of hypotension and bradycardia could be expected more frequently with hyperbaric prilocaine. Indeed, despite similar incidence of hemodynamic side effects between groups, the absence of bradycardia with chloroprocaine is noteworthy. Similarly, Casati et al*.* reported one event of hypotension and no bradycardia with 50 mg of chloroprocaine [22]. On the contrary, bradycardia and hypotension seem to be regularly recorded with hyperbaric prilocaine: Manassero et al. reported 9 cases of hemodynamic side effects with 80 patients [12].

In the present work chloroprocaine and hyperbaric prilocaine failed to provide adequate anesthesia for 7.6% of the patients with hyperbaric prilocaine and 5.1% with chloroprocaine. Spinal anesthesia failures are described in other studies but only two of these studies used 50 mg of both local anesthetics. Moreover, surgeries are not restricted to the lower limb [5, 8, 20, 23] Hendricks et al.recorded 5.5% of failure with hyperbaric prilocaine [6]. Casati et al. did not recorded any patients who required supplementary analgesia during surgical procedures with 50 mg of chloroprocaine for lower limb surgery [22].

We did not observe any TNS. Indeed, only one TNS has been reported with chloroprocaine [24]. TNS is also notably rare for hyperbaric prilocaine: König et al*.* did not record any case in the retrospective analysis of 5000 spinal anesthetics with the use of plain prilocaine [25, 26]

The major limitation of our study is the dose of chloroprocaine. Previous works suggested that 50 mg of hyperbaric prilocaine is the optimal clinical dose for knee arthroscopy [8, 11]. Doses ranging from 20 to 60 mg of chloroprocaine have been described and multiple comparisons between chloroprocaine and other local anesthetics have been performed [19, 27–29]. In a study with increasing doses of chloroprocaine (30–40-50 mg) Casati et al*.* described inadequate anesthesia for lower limb surgery with 30 and 40 mg, this was not the case with 50 mg [22]. Nevertheless, ED_95_ of chloroprocaine for knee arthroscopy has still to be defined. Indeed, comparisons of drugs are highly affected by the chosen doses: Wesselink et al*.* compared 40 mg of chloroprocaine with 40 mg of hyperbaric prilocaine in term of durations of motor block. Compared to the present study, the dose of the short acting chloroprocaine was 10 mg less; this was also the case for the intermediate acting hyperbaric prilocaine that was used with a dose corresponding to the ED_90_ [11, 30]. In these conditions, mean duration of motor block was 15 min shorter for chloroprocaine compared to hyperbaric prilocaine. Moreover, decreasing the dose of hyperbaric prilocaine to the ED_90_ was probably related to the absence of motor block for 9.3% of the patients.

## Conclusion

Considering the time to full recovery of sensory block, chloroprocaine may result in an earlier discharge of patients compared to hyperbaric prilocaine. Nevertheless, the choice of local anesthetic should not be determined only by the duration of spinal anesthesia but also by the dermatome level required for the type of surgery. Altogether, the differing properties of these two local anesthetics and the low incidence of side effects observed with both drugs, allow the anesthesiologist to fine-tune spinal anesthesia in the ambulatory surgery setting.

## Data Availability

The datasets used and/or analysed during the current study are available from the corresponding author on reasonable request.

## References

[1] Freedman JM, Li DK, Drasner K (1998). Transient neurologic symptoms after spinal anesthesia: an epidemiologic study of 1863 patients. Anesthesiology.

[2] Zaric D, Pace NL (2009). Transient neurologic symptoms (TNS) following spinal anesthesia. Cochrane Database Syst Rev.

[3] Malinovsky JM (2012). Is 4% articaine suitable for spinal anesthesia?. Eur J Anesthesiol.

[4] Molenberghs G, Verbeke G (2005). Models for discrete longitudinal data.

[5] Förster JG, Rosenberg PH (2013). Chloroprocaine 40 mg produces shorter spinal block than articaine 40mg in day-case knee arthroscopy patients. ActaAnesthesiolScand.

[6] Hendriks MP, de Weert CJ, Snoeck MM (2009). Plain articaine or prilocaine for spinal anesthesia in day-case knee arthroscopy: a double-blind randomized trial. Br J Anesth.

[7] Förster JG, Rosenberg PH (2011) Revival of old local anesthetics for spinal anesthesia, in ambulatory surgery. CurrOpinAnesthesiol 24:633–637 10.1097/ACO.0b013e32834aca1b 21841475

[8] Guntz E (2016). Choosing the best local anesthestic for spinal anesthesia. Reg Anesth Pain Med.

[9] Förster JG, Kallio H, Rosenberg PH et al (2011) Chloroprocaine vs. Articaine as spinal anesthetics for day-case knee arthroscopy. Acta Anesthesiol Scand 55:273–281 10.1111/j.1399-6576.2010.02325.x 21039353

[10] Casati A, Fanelli G, Danelli G (2007). Spinal anesthesia with lidocaine or preservative-free 2-chlorprocaine for outpatient knee arthroscopy: a prospective, randomized, double-blind comparison. AnesthAnalg.

[11] Guntz E, Latrech B, Tsiberidis C (2014). ED50 and ED90 of intrathecal hyperbaric 2% prilocaine in ambulatory knee arthroscopy. Can J Anesth.

[12] Manassero A, Bossolasco M, Ugues S (2014). Comparison of unilateral and bilateral spinal anesthesia with 2% hyperbaric prilocaine in day-case inguinal herniorrhaphy: a randomized controlled trial. Minerva Anestesiol.

[13] Rätsch G, Niebergall H, Hauenstein L (2007). Spinal anesthesia in day-case surgery. Optimisation of procedures. Anesthesist.

[14] Förster JG (2014). Short-acting spinal anesthesia in the ambulatory setting. CurrOpinAnesthesiol.

[15] Guntz E, Kapessidou Y (2016). Spinal prilocaine for same-day surgery: the importance of equipotent doses. Can J Anesth.

[16] Kreutziger J, Frankenberger B, Luger TJ (2010). Urinary retention after spinal anesthesia with hyperbaric prilocaine 2% in an ambulatory setting. Br J Anesth.

[17] Aguirre J, Borgeat A, Bühler P (2015). Intrathecal hyperbaric 2% prilocaine versus 0.4% plain ropivacaine for same-day arthroscopic knee surgery: a prospective randomized double-blind controlled study. Can J Anesth.

[18] Camponovo C, Fanelli A, Ghisi D (2010). A prospective, double-blinded, randomized, clinical trial comparing the efficacy of 40 mg and 60 mg hyperbaric 2% prilocaine versus 60 mg plain 2% prilocaine for intrathecal anesthesia in ambulatory surgery. AnesthAnalg.

[19] Hejtmanek MR, Pollock JE (2011). Chloroprocaine for spinal anesthesia: a retrospective analysis. ActaAnesthesiolScand.

[20] Breebaart MB, Teune A, Sermeus LA (2014). Intrathecal chloroprocaine vs lidocaine in day-case surgery: recovery, discharge and effect of pre-hydration on micturition. ActaAnesthesiolScand.

[21] Mulroy MF, Salinas FV, Larkin KL (2002). Ambulatory surgery patients may be discharged before voiding after short-acting spinal and epidural. Anesthesia Anesthesiology.

[22] Casati A, Danelli G, Berti M (2006). Intrathecal 2-chloroprocaine for lower limb outpatient surgery: a prospective, randomized, double-blind, clinical evaluation. AnesthAnalg.

[23] Ambrosoli AL, Chiaranda M, Fedele LL (2016). A randomised controlled trial of intrathecal blockade versus peripheral nerve blockade for day-case knee arthroscopy. Anesthesia.

[24] Lacasse MA, Roy JD, Forget J (2011). Comparison of bupivacaine and 2-chloroprocaine for spinal anesthesia for outpatient surgery: a double-blind randomized trial. Can J Anesth.

[25] Hampl KF, Heinzmann-Wiedmer S, Luginbuehl I (1998). Transient neurologic symptoms after spinal anesthesia: a lower incidence with prilocaine and bupivacaine than lidocaine. Anesthesiology.

[26] Konig W, Ruzicic D (1997). Absence of transient radicular irritation after 5000 spinal anesthetics with prilocaine. Anesthesia.

[27] Davis BR, Kopacz DJ (2005). Spinal 2-chloroprocaine: the effect of added clonidine. AnesthAnalg.

[28] Kopacz DJ (2005). Spinal 2-chloroprocaine: minimum effective dose. RegAnesth Pain Med.

[29] Smith KN, Kopacz DJ, McDonald SB (2004). Spinal 2-chloroprocaine: a dose-ranging study and the effect of added epinephrine. AnesthAnalg.

[30] Wesselink E, Hurk GJD (2019). Chloroprocaine versus prilocaine for spinal anesthesia in ambulatory knee arthroscopy: a double-blind randomized trial. RegAnesth Pain Med.

